# Cardiovascular and Autonomic Dysfunction in Murine Ligature-Induced Periodontitis

**DOI:** 10.1038/s41598-020-63953-1

**Published:** 2020-04-23

**Authors:** Aline Barbosa Ribeiro, Nilton Nascimento Santos-Junior, João Paulo Mesquita Luiz, Mauro de Oliveira, Alexandre Kanashiro, Thaise Mayumi Taira, Sandra Yasuyo Fukada, José Carlos Alves-Filho, Rubens Fazan Junior, Helio Cesar Salgado

**Affiliations:** 1 Department of Physiology, Ribeirão Preto Medical School. University of São Paulo. Ribeirão Preto, São Paulo, Brazil; 2 Department of Pharmacology, Ribeirão Preto Medical School, University of São Paulo, Ribeirão Preto, SP Brazil; 3 Department of Neurosciences and Behavior, Ribeirão Preto Medical School, University of São Paulo, Ribeirão Preto, SP Brazil; 4 Department of Bio Molecular Sciences, School of Pharmaceutical Sciences of Ribeirao Preto, University of São Paulo, Ribeirão Preto, Brazil

**Keywords:** Cardiology, Diseases, Risk factors

## Abstract

The present study examined the hemodynamics [arterial pressure (AP), AP variability (APV), heart rate (HR), and heart rate variability (HRV)], cardiac function (echocardiographycally), and myocardial inflammation in Balb/c mice submitted to Periodontitis, through the ligation of the left first molar, or Sham surgical procedure. The first protocol indicated that the AP was similar (136 ± 2 vs. 132 ± 3 mmHg in Sham), while the HR was higher in mice with Periodontitis (475 ± 20 vs. 412 ± 18 bpm in Sham), compared to their Sham counterparts. The APV was higher in mice with Periodontitis when evaluated in the time domain (4.5 ± 0.3 vs. 3.4 ± 0.2 mmHg in Sham), frequency domain (power of the LF band of systolic AP), or through symbolic analysis (patterns 0V + 1V), indicating a sympathetic overactivity. The HRV was similar in the mice with Periodontitis, as compared to their Sham counterparts. In the second protocol, the mice with Periodontitis showed decreased cardiac output (10 ± 0.8 vs. 15 ± 1.4 mL/min in Sham) and ejection fraction (37 ± 3 vs. 47 ± 2% in Sham) associated with increased myocardial cytokines (Interleukin-17, Interleukin-6, and Interleukin-4). This study shows that experimental Periodontitis caused cardiac dysfunction, increased heart cytokines, and sympathetic overactivity, in line with epidemiological studies indicating an increased risk of cardiovascular events in clinical Periodontitis.

## Introduction

Periodontitis is caused by a chronic inflammatory response to a periodontal biofilm that deranges the supporting tissues around the teeth (alveolar bone, periodontal ligament, and cementum), causing dental loss^1^. This inflammatory disease represents a major public health problem, with an estimated prevalence of over 790 million people worldwide^2,3^.

Of note, more than 700 species of bacteria are estimated in the oral cavity forming the dental biofilm^4,5^. Biofilms can release biologically active products, including bacterial lipopolysaccharides, chemotactic mediators, protein toxins, and organic acids. These active products elicit different components of innate and adaptive immunity^6^ followed by the production of inflammatory mediators, mainly cytokines produced by immune cells^7^. These cytokines, including interleukin (IL)-17, IL-6, IL-4, and other inflammatory mediators, such as C-reactive protein, are released in response to the stimuli induced by the dental biofilm^8^.

Different rodent models of periodontal disease have been described to investigate the pathophysiology of this inflammatory disease and accompanying complications^9,10^. Nevertheless, the placement of a ligature around a molar tooth is one of the most widely used model^11^. Indeed, ligatures around the posterior teeth mimic human periodontitis, leading to local inflammatory cell accumulation, apical migration of junctional epithelium, and bone loss^9,12,13^. Moreover, the structure of the dental gingival area in the rodent is quite similar to that exhibited by humans^14^.

Recent studies have proven that the harmful effects of periodontitis are not restricted to the oral cavity, but can also affect the overall health of the subjects^15^. A growing body of evidence has, epidemiologically, associated the periodontal inflammation with cardiovascular diseases, such as arterial hypertension, myocardial infarction, stroke, and atherosclerotic vascular disease^16–19^. Besides, it has been postulated that this relationship can be both, indirect, i.e., via shared risk factors^20^, and direct, i.e., through oral bacteria translocation to the bloodstream eliciting a generalized inflammatory response^21^. However, the mechanisms of the relationship between periodontitis and cardiovascular disease are not entirely understood.

Our group has demonstrated that the oscillatory patterns of cardiovascular parameters, such as heart rate (HR) and arterial pressure (AP), are widely used to evaluate the neuro-humoral control of the cardiovascular system in inflammatory conditions^22^. In addition, it is also known that changes in heart rate variability (HRV), an important indicator of autonomic function, are predictors of morbidity and mortality in different cardiovascular and systemic diseases^23,24^. In fact, impaired HRV indices had already been demonstrated in patients with periodontitis^25^, which corroborates with possible association between oral diseases and cardiovascular risk factors. However, the effect of periodontitis in HRV and APV was never been evaluated experimentally.

Since the role of periodontal disease eliciting cardiovascular dysfunctions has been poorly investigated, the purpose of this study was, therefore, to assess the cardiovascular consequence in a murine model of inflammatory gingival process. Thus, the goal of the current research was achieved using several approaches such as echocardiographic evaluation of cardiac performance, myocardial quantification of inflammatory cytokines, as well as hemodynamic (arterial pressure and heart rate variability) recordings were employed.

## Material and Methods

### Animals

Adult male Balb/c mice (22–26 g) were obtained from the breeding facility of the University of São Paulo at Ribeirão Preto. The animals were housed under a 12 h light/dark cycle and controlled temperature (24 ± 2 °C). Food and water were provided *ad libitum*. All procedures adhered to the “Guide for the Care and Use of Laboratory Animals” prepared by the National Academy of Sciences and published by the National Institutes of Health (National Academy of Sciences, 1996), and were approved by the Ethics Committee of Ribeirão Preto Medical School, University of São Paulo, São Paulo, Brazil (protocol number 231/2018).

### Surgical procedures

Under ketamine (100 mg/kg) and xylazine (10 mg/kg), intraperitoneal anaesthesia, Balb/c mice were submitted to the ligation of the mandibular left first molar with 4–0 sterile silk suture for periodontitis induction. Control animals were subjected to Sham operation. After 25 days, under inhalation of isoflurane anaesthesia (5% induction, 2–3% maintenance), mice were implanted with a Micro-Renathane tubing catheter into the femoral artery for direct AP measurement. All animals received penicillin/streptomycin (200 IU/g body weight and 80 μg/g body weight, respectively, i.m.) and were allowed to recover in individual cages.

### Experimental protocol

Five days after the surgical procedure, the catheter was connected to a pressure transducer (Model DPT-100 Deltran; Utah Medical Products, Midvale, UT, USA) attached to an analogic to the digital interface (PowerLab 4/40, ADInstruments, Australia). After at least 30 min, necessary for the adaptation of the animal to the recording system, the AP was continuously sampled (2 kHz) in an IBM/PC, during 40 min, and stored for offline analysis.

Two distinct groups of mice had their cardiac function assessed by echocardiography, thirty days after the dental ligation, or Sham operation. After the echocardiographic examination, these animals were sacrificed by an anaesthetic overdose and had their jaw carefully collected, and fixed with 10% formalin. Besides, these mice had their chest opened, and the heart quickly removed, rinsed with cold saline, and frozen at −80 °C for measuring the myocardial cytokines.

### Evaluation of alveolar bone loss

The jaws were immersed in 9% hypochlorite for 5 hours to remove soft tissues, and the samples were stained with 1% methylene blue (1 g/100 mL, diluted in water) for 5 min. The distance and area between the cementoenamel junction and alveolar bone crest were measured using a microscope (D.F. Vasconcellos, Brazil). A digital camera was used to determine the alveolar bone loss, and StCamSWare 1.1 captured the images. Measurement of the distance (average of the three points)^26^ and area between the cementoenamel junction and the alveolar bone crest on the buccal and lingual surfaces was achieved, and values were calculated using the Image J (National Institutes of Health, USA) computer software. The measurements in pixels were converted into millimetres using the markings of the ruler to which the jaw was attached as a reference.

### Analysis of AP and HR variability

Beat-by-beat time series of systolic AP and pulse interval (PI) values were detected, and calculated from pulsatile AP recordings by a specially designed computer software (Blood Pressure Module for LabChart, ADInstruments, Australia). All indices of variability were calculated with the custom-made computer software, developed in our laboratory, PhyBios.

#### Time-domain analysis

HRV was assessed in time-domain by the calculation of the standard deviation of normal-to-normal PI values (SDNN), and the root mean square of successive differences between PI (RMSSD). Blood pressure variability was assessed only by the standard deviation (SD) of the systolic values of AP.

#### Spectral analysis (Frequency Domain)

beat-by-beat series of PI and systolic AP values were resampled to 10 Hz by cubic spline interpolation and divided into half-overlapping segments of 512 points (51.2 s). Following, a Hanning window was applied to each segment, and spectra were calculated by Fast Fourier Transform (FFT) and integrated into low- (LF: 0.2 to 1.0 Hz) and high-frequency bands (HF: 1.0 to 5 Hz). The power of the spectra in LF band is shown in normalized units (nu) while in the HF band is shown in absolute units (ms^2^ or mmHg^2^)^27^. Normalized values were obtained following calculation of the percentage of LF and HF power apropos of total power, pertinent to the total power of the spectrum minus the power at the very-low-frequency band (VLF: <0.02 Hz)^28^.

### Symbolic analysis of HRV

Symbolic analysis was performed as described elsewhere^29,30^. Briefly, the range of values of the PI series was divided into six equally spaced segments; and the values were transformed into symbols (from 0 to 5), they belong to according to the segment. Following, a combination of 3 consecutive symbols were analysed, and classified into one of the following patterns: 0V, sequence with no variation, when all three symbols were equal; 1V, sequence with one variation, when two consecutive symbols were identical, and the remnant was different; and 2V, sequence with two variations, when all the 3 symbols are different. Sequences with 2 variations were additionally classified in 2LV when the 2 changes are in the same direction (increasing, or decreasing), and 2UV when the changes are in the opposite direction. The percentage of patterns 0V and 2UV was computed for the whole series^29,30^. The fundament of the variation patterns built is based on different characteristics of autonomic modulation of the heart, i.e., slow modulation of sympathetic (less variation of PI intervals in a certain period of time) and fast modulation of parasympathetic (more variation of PI in a certain period of time). Therefore, 0V pattern occurrence reflects sympathetic modulation, while the 2V pattern reflects vagal modulation^30^.

In the case of systolic AP, only 0V and 1V sequences were computed together and were considered an index of the sympathetic modulation of the arterial vessels^31^.

### Echocardiography

Echocardiography was performed in two distinct groups of mice, around 30 days after ligature, with a Vevo 2100 High-Resolution Imaging System (Visual Sonics, Toronto, ON, Canada), using a 30-MHz transducer. The steps and procedures were performed as previously described^32^. Briefly, mice were slightly anaesthetized using 1.5% isoflurane in 100% oxygen. After reaching the appropriate level of anaesthesia, each mouse was placed in the dorsal decubitus for two-dimensional M-mode image acquisition. The body temperature was monitored during all procedure. Cardiac output, ejection fraction and fractional shortening were measured during systole and diastole, offline, from parasternal long-axis images.

### Cytokines measurement

Myocardial samples were thawed and maintained on ice. The heart was homogenized in 0.5 mL of PBS and then centrifuged at 3500 rpm for 15 min at 4 °C. The cytokines (IL-4, IL-6, and IL-17) were measured in the supernatants, using appropriate ELISA kits (R&D Systems, Minneapolis, Minn., USA) according to the manufacturer’s instructions. The lower limit of the kits for the detection of IL-4, IL-6, and IL-17 was 16 pg/mL. The results of the cytokine concentration in the supernatant of the heart tissue are expressed as pg/mg of tissue weight.

### Statistical analysis

The statistical analysis was performed using unpaired Student’s *t-*test. Values are expressed as the mean ± SEM. Differences were considered significant at *P* < 0.05.

## Results

### Comparison of attachment and alveolar bone loss

**Figure 1:**
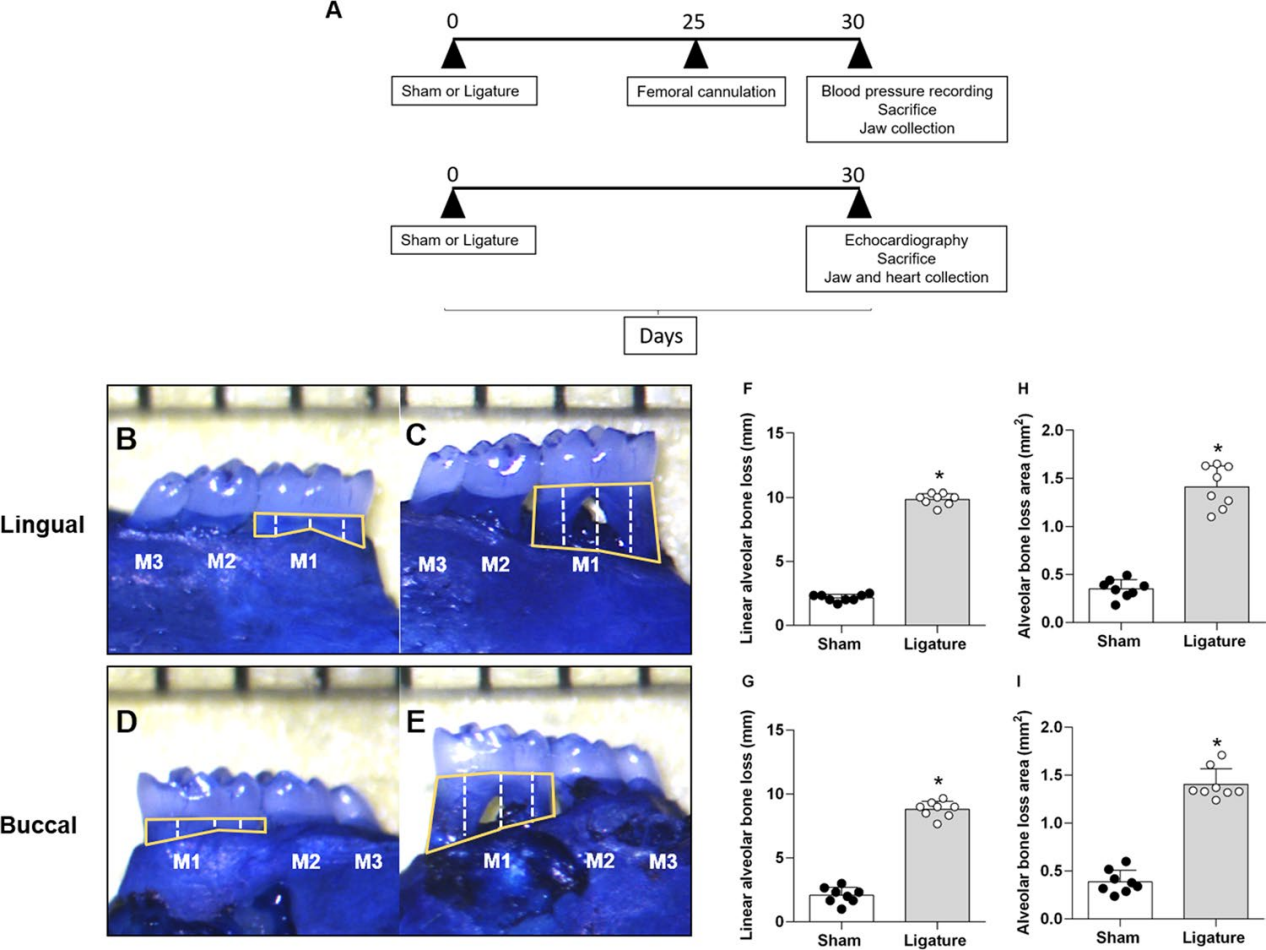
Effect of periodontitis in alveolar bone loss. (**A**) Protocol of experiments of mice ligature periodontitis model. Linear and area of the alveolar bone loss was measured macroscopically in the lingual (Panels B,C) and buccal (Panels D,E) surfaces in mice with Sham (Panels B,D) or dental ligation (Panels C,E). Bar graphs show the linear alveolar bone loss and alveolar bone loss area from the lingual (Panel F,H, respectively) and buccal (Panel G,I, respectively) surfaces. The white dashed lines indicate the three distances measured in the teeth and yellow solid lines indicate alveolar bone loss area. Data are mean ± SEM *P < 0.05. Ligature: Periodontitis elicited by the ligation of the mandibular left first molar. Sham: Sham ligation.

Alveolar bone resorption is the hallmark of periodontitis induced by the ligature. Figure 1 shows the comparison of morphometric measurements between ligature-induced periodontitis mice and Sham mice. Buccal distances and area from the cementoenamel junction to the alveolar bone crest were significantly higher in the ligature-induced periodontitis subjects as compared with their control (Sham) counterparts. Robust bone loss was also observed on lingual side evidenced by increased distances and area between cementoenamel junction to alveolar bone crest in the ligature-induced periodontitis mice when compared with their Sham-operated counterparts.

### Variability of systolic AP and PI

**Figure 2:**
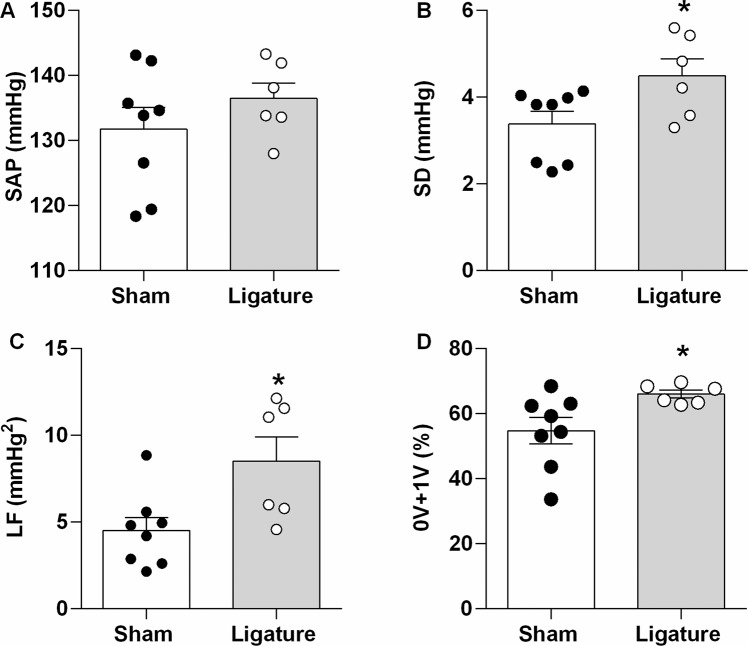
Systolic Arterial Pressure Variability in Time and Frequency Domain, and Symbolic Dynamics Analysis. Bar graphs show in the Upper Panels the systolic arterial pressure (SAP; Panel A) and the standard deviation of SAP (SD; Panel B); and, in the Lower Panels the power of the low-frequency component of heart rate variability (LF; Panel C), and Families from the Symbolic Dynamics Analysis (0V + 1V Families; Panel D). Data are mean ± SEM. *P < 0.05. Ligature: Periodontitis elicited by the ligation of the mandibular left first molar. Sham: Sham ligation.

Figure 2 shows the systolic AP and the indices of variability from mice with ligature-induced periodontitis and their control (Sham) counterparts. The mean systolic AP is similar between both groups; nevertheless, periodontitis elicited a higher overall systolic APV in the time domain (SD of systolic AP values). Moreover, the power of the LF band of systolic AP and the occurrence of patterns 0V + 1V in the symbolic analysis (both indices of cardiac sympathetic modulation), were higher in mice with ligature-induced periodontitis, compared with the Sham-operated counterparts.

**Figure 3:**
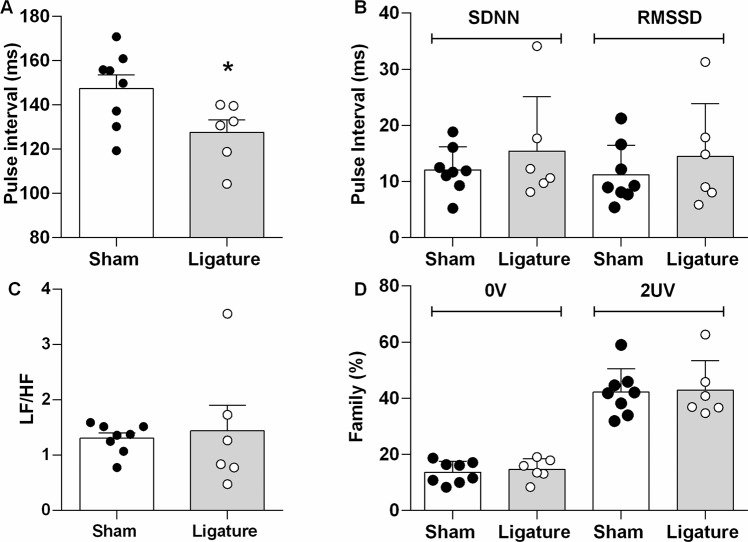
Heart Rate Variability in Time and Frequency Domain, and Symbolic Dynamics Analysis. Bar graphs show: Panel A, Mean Pulse Interval; Panel B, Standard Deviation of the NN interval (SDNN) and The Square Root of the Mean Squared Differences of Successive NN Intervals (RMSSD) from the Pulse Interval; Panel C, The ratio between the powers of Low Frequency (LF) and High Frequency (HF) components of Heart Rate Variability; Panel D, Families (0V and 2UV) from Symbolic Dynamics Analysis of Pulse Interval. Data are mean ± SEM. *P < 0.05. Ligature: Periodontitis elicited by ligation of the mandibular left first molar. Sham: Sham ligation.

Figure 3 shows the heart rate variability in time and frequency domain and symbolic dynamics analysis of the PI. The mean PI was shorter in mice with ligature-induced periodontitis, compared with their control Sham-operated subjects. Overall, the PI variability in the time domain, characterized by SDNN and RMSSD, and the HRV calculated from power spectral and symbolic analysis were similar between both groups.

### Cardiac function

**Figure 4:**
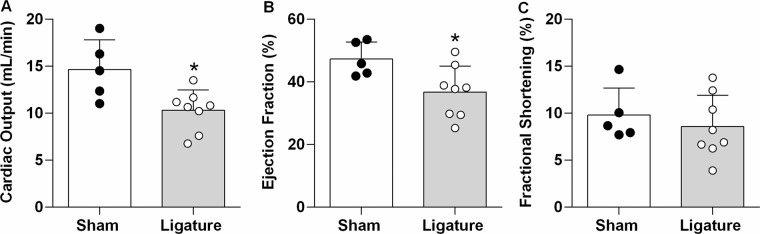
Cardiac Function in Periodontitis Elicited by Ligation of Mandibular Left First Molar. Bar Graphs show: Panel A, Cardiac Output; Panel B, Ejection Fraction; Panel C, Fractional Shortening. Data are mean ± SEM. *P < 0.05. Ligature: Periodontitis elicited by ligation of the mandibular left first molar. Sham: Sham ligation.

The assessment of cardiac function (Fig. 4) shows that both the cardiac output and ejection fraction were reduced in mice with ligature-induced periodontitis. However, the fractional shortening was similar in both groups.

### Cytokines in the heart

**Figure 5:**
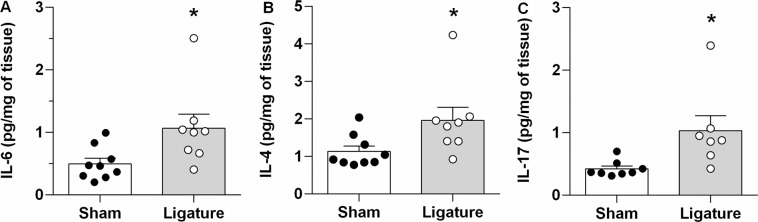
Inflammatory Cytokine Concentration in the Cardiac Tissue, in Periodontitis Elicited by Ligation of Mandibular Left First Molar. Bar Graphs show: Panel A, interleukin-6 (IL-6); Panel B, interleukin-4 (IL-4); Panel C, IL-17. Data are mean ± SEM. *P < 0.05. Ligature: Periodontitis elicited by ligation of the mandibular left first molar. Sham: Sham ligation.

The concentration of IL-6, IL-4 and IL-17 in the heart was assessed to examine the inflammatory response elicited by periodontitis (Fig. 5). The levels of cytokines were higher in mice with ligature-induced periodontitis, compared with their Sham-operated controls.

## Discussion

In the present study, the bone loss found in mice subjected to dental ligation showed that the model of periodontitis examined was straightforward. The evaluation of the hemodynamics (HR and APV), cardiac function (echocardiography), and concentrations of myocardial cytokines revealed the impact of periodontitis in the cardiovascular system in this experimental model of periodontitis.

The basal levels of AP were not different between periodontitis and Sham groups. However, among the principal findings of the current study, it is of note the high overall variability of AP in mice with gingival infection. Several pieces of evidence have shown an association between arterial pressure variability and cardiovascular diseases, independently of the basal arterial pressure levels^33–35^. Clinically, high arterial pressure variability is often associated with dysfunction of the baroreflex and autonomic imbalance, characterized by sympathetic overactivity, leading to an increased risk of life-threatening cardiovascular events^34,36^. Deleterious events related to high arterial pressure variability occur either, during hypotensive situations leading to coronary hypoperfusion^37^ or when blood pressure rises, leading to myocardial dysfunction, ventricular hypertrophy, and stroke^38^. However, the mechanisms through which the arterial pressure variability causes organ damage, in the absence of arterial hypertension, are still a matter of debate.

In addition to the increase in overall APV, the mice with periodontitis also showed high values of the AP spectra at the low-frequency (LF) band and increased incidence of 0V sequences in the symbolic analysis. Either, spectral analysis (linear) or symbolic dynamics (non-linear), are approaches based on APV that provide information regarding autonomic vasomotor modulation^30,31^. Therefore, the data from spectral analysis (linear) and symbolic dynamics (non-linear) obtained in mice with gingival infection are reliable indicators of vascular sympathetic overactivity.

The basal tachycardia presented by mice with periodontitis corroborates the hypothesis of sympathetic overactivation. It is well established that sustained sympathetic overactivity has been associated with the development of end-organ damage, such as cardiac hypertrophy and deterioration of kidney function^39^. In this context, the finds of the current study support the notion of increased cardiovascular risk associated with gingival infection in the model evaluated. Surprisingly, besides the shorter basal PI (tachycardia) found in mice with experimental periodontitis, all HRV indices were found similar in both groups evaluated. Segovia *et al*. (2012) showed the reduction of HRV indices in the time domain (SDNN and RMSSD), in patients with periodontitis compared to healthy individuals^25^. However, similar to the present study, these findings suggest a sympathetic overactivity induced by periodontitis, but in HRV indices^25^ and not in APV indices. Interestingly, this study did not show the HR, PI or APV values obtained from these patients. It has been well documented that both HRV and APV are reliable indices expressing the modulation of the autonomic function^23,24,34,36,38^.

Moreover, the echocardiographic approach evidenced lower cardiac output and ejection fraction, indicating an impairment of left ventricular function in mice with ligature-induced periodontitis. In line with data from HR and APV, changes in cardiac function found in the present study provide support to the notion that oral diseases can derange a healthy heart. Moreover, despite limitations in clinical research, studies have shown the association between the severity of periodontitis and cardiac hypertrophy in humans^40,41^. Besides, experimental studies with the administration of *Porphyromonas gingivalis*, one of the major bacteria involved in periodontitis, demonstrated the development of cardiac hypertrophy and fibrosis^42,43^. In addition, in the experimental model of *Aggregatibacter actinomycetemcomitans* infection, there was also an increase in myocardial hypertrophy, fibrosis, and arteriosclerosis in the transverse aortic constriction model^42^.

Importantly, in addition to the histopathological findings, we observed in the current study that ligature-induced periodontitis showed the increased concentrations of the cytokines IL-17, IL-6, and IL-4 in the heart of mice with gingival inflammation. The presence of these cytokines, especially IL-17 and IL-6, are strongly associated with cardiovascular alterations^8,44,45^. Corroborating these findings, other experimental studies in mice have also shown high levels of inflammatory mediators in the heart, following systemic administration of bacterial components or periodontal bacteria^42,46–51^, although few studies evaluated the heart cytokines in ligature model. These inflammatory mediators could cause derangement of the heart and autonomic dysfunction; for instance, IL-17 has been crucial to myocarditis caused by *Porphyromonas gingivalis*^46^. Other cytokines such as TNF-α, TGF-β, IL-1 IL-4, IL-6, IL-8, and IL-18 are also related to the development of inflammatory pathologies involving the heart (ischemic heart disease, myocardial infarction, heart failure, and cardiomyopathies)^52^. However, further studies are needed to elucidate the role of the autonomic nervous system (sympathetic and parasympathetic) in the modulation of these cardiac cytokines induced experimental periodontitis.

Previous studies suggested that potential mechanisms linking periodontitis and cardiac dysfunction include the direct effects of bacteria and the indirect effects through host inflammatory responses. Invasion of bacteria on endothelial cells, monocytes and cardiovascular tissue have been reported; while the impact on the cardiovascular system seems biologically reasonable^53–55^. However, several lines of evidence indicate that the effect of periodontal disease in cardiovascular events is more related to host inflammatory responses than the direct impact of the periodontopathic bacteria themselves. Inflammatory cytokines can promote cell adhesion, increased permeability, and apoptosis by interacting with specific receptors on different cell types^56^. Likewise, pro-inflammatory cytokines induced by periodontitis can also act indirectly, increasing reactive oxygen species, which can induce oxidative stress locally or in distant organs^57^.

Accumulating evidence has indicated that inflammation causes increased activity of the sympathetic nervous system with the release of noradrenaline and neurotransmitters in lymphoid organs and inflamed local sites^58,59^. Therefore, chronic activation of the sympathetic nervous system by changing the role of immune cells contributes to fibrosis and hypertrophy of the heart, deranging the heart function and APV^60^. Of note, the concept of neuroimmunomodulation has emerged from studies of dynamic interactions between the nervous and immune systems in non-periodontal disease mediated by cytokines^61–63^. As a result, it can be predicted that the systemic spillover of cytokines during periodontal disease would engage the sympathetic nervous system exacerbating cardiac inflammation, leading to alterations of the cardiovascular indices.

On top of that, studies are needed to elucidate: (1st) whether the hyperactivity of cardiac sympathetic innervations, promoted by periodontal inflammation, determines the inflammatory response of the heart; (2nd) whether the local inflammation triggers a specific neural reflex eliciting the activation of the sympathetic nervous system. Apropos, it has been demonstrated that increased vascular sympathetic activity results in the mobilization of the hematopoietic stem cells to the arteries, causing vascular inflammation that promotes atherosclerosis^64^. On the other hand, several studies have demonstrated that autonomic neuro-immune signalling exerts an immediate and specific anti-inflammatory response^61–63^ or contributes to the hypertension^65^. Moreover, further studies are also needed to confirm whether this brain-heart neural circuit is activated to improve the organ-specific inflammatory response in the heart or vice-versa.

Current strategies for treating periodontitis aim to limit its influence in local and systemic inflammation. New approaches based on the regenerative medicine by the development of new biomaterials from the oral-derived mesenchymal stem cells and novel biomimetic scaffolds in dental repairing, have been identified^66–68^. These strategies aim to induce not only a reparative process but also an immunomodulatory activity, improving the reparative process and the support of scaffolds to dental implants.

In conclusion, to our knowledge, this is the first study that explored the effects of periodontal inflammation on the cardiovascular system at an experimental level. The findings of this study corroborate with clinical evidence and provide support to the notion that periodontitis can affect the cardiovascular system promoting inflammatory response^16–19^. Therefore, more studies that are conspicuous are necessary to deeply evaluate the mechanisms involved in this complex neuro-immune-heart circuit. Nevertheless, the findings observed in this study highlight the importance of this multi-systems interaction in the association between periodontitis and the epidemiological risk of cardiovascular events.
